# Prostate Cancer Screening in Contemporary Era: PSA-Based Testing and Risk-Adapted Approaches

**DOI:** 10.3390/cancers18101547

**Published:** 2026-05-10

**Authors:** Michele Brancaccio, Armando Galdieri, Andrea Cosenza, Francesco Barletta, Pietro Scilipoti, Leonardo Quarta, Paolo Zaurito, Alfonso Santangelo, Alessandro Viti, Angelo Occhi, Maria Elena Porzi, Alessia Colistro, Giulia Roca, Simone Scuderi, Vito Cucchiara, Armando Stabile, Francesco Montorsi, Alberto Briganti, Giorgio Gandaglia

**Affiliations:** 1 Division of Urology, IRCCS San Raffaele Scientific Institute, 20132 Milan, Italy; 2 Vita-Salute San Raffaele University, 20132 Milan, Italy

**Keywords:** prostate cancer, screening, prostate-specific antigen, magnetic resonance imaging, polygenic risk scores, active surveillance

## Abstract

Prostate cancer (PCa) screening has long relied on PSA testing, a strategy that has shaped diagnostic pathways for decades but remains limited by substantial overdiagnosis and downstream overtreatment. As screening practices evolve, the relevance of historical evidence must be reconsidered in the context of contemporary diagnostic workflows that now incorporate imaging, refined biopsy techniques, and risk-adapted management. This narrative review examines the transition from PSA-only screening toward modern, risk-adapted early detection strategies. We synthesize evidence supporting the integration of mpMRI, refined biopsy techniques, and active surveillance (AS) as key components of contemporary screening pathways aimed at improving the detection of clinically significant disease while minimizing unnecessary interventions. Particular emphasis is placed on second-line MRI-based approaches, which consistently reduce the number of avoidable biopsies and enhance the diagnostic precision. In addition, we review the role of blood-based and genomic biomarkers in pre-biopsy risk stratification, discussing established tools within a unified framework of individualized screening. The review also contextualizes very recent regulatory developments, including the FDA approval of a novel structure-based PSA assay, as part of the ongoing evolution of biomarker-supported screening rather than a paradigm shift. Overall, this article provides a timely synthesis of mature randomized evidence and emerging diagnostic innovations, offering a clinically grounded perspective on how PCa screening is being reshaped toward more personalized and harm-aware strategies.

## Simple Summary

For many years, the early detection of prostate cancer (PCa) has relied mainly on the prostate-specific antigen (PSA) blood test. Although this test has improved the diagnostic pathway for PCa, it has also led to the diagnosis and treatment of indolent tumors. Today, in addition to PSA, multiparametric magnetic resonance imaging (mpMRI) is used to better identify more aggressive cancers that truly need to be treated. Novel blood tests and genetic biomarkers may also help to estimate the individual risk of PCa before deciding on biopsy, while active surveillance (AS) represents a key strategy for low-risk disease that does not require immediate treatment but can instead be safely monitored over time. Overall, PCa screening is evolving toward safer, more tailored strategies that aim to detect clinically significant disease while minimizing the overdiagnosis of indolent disease and subsequent unnecessary treatments.

## 1. Introduction

PCa is one of the most commonly diagnosed malignancies worldwide [1]. The incidence and mortality are projected to increase over the coming years, representing a growing healthcare challenge [1]. Given this epidemiological impact, the implementation of effective screening strategies remains paramount for early disease control. The main purpose of screening is the early detection of clinically significant tumors at a potentially curable stage, thereby reducing cancer-related mortality. PSA, in combination with digital rectal examination (DRE), has long represented the cornerstone of PCa early detection. PSA is a serine protease secreted by prostatic epithelial cells and is physiologically involved in the liquefaction of the seminal coagulum to promote sperm motility. However, PSA serum levels may be elevated not only in PCa but also in other conditions, such as benign prostatic hyperplasia and prostatitis. Despite its limited specificity, PSA has served as the main tumor marker for PCa screening for more than three decades [2]. Over time, numerous new strategies have been explored to overcome the limitations of PSA-based screening. Among these, MRI has emerged as a pivotal tool in the contemporary management of PCa, due to its ability to enable the detailed anatomical and functional characterization of the index prostatic lesion. The development of standardized mpMRI protocols, combining anatomical sequences of T1-weighted images (T1WI) and multiplanar T2-weighted images (T2WI) with functional sequences including dynamic contrast enhancement (DCE) and diffusion-weighted imaging (DWI), has significantly improved PCa detection, local staging, and treatment planning [3]. More recently, abbreviated non-contrast protocols, such as biparametric MRI (bpMRI), have been introduced. This imaging modality, which omits the DCE sequence, offers shorter acquisition times, improved cost-effectiveness, and the avoidance of contrast-related adverse events, while maintaining adequate diagnostic performance [4]. In the screening setting, MRI holds the potential to enhance risk stratification in men suspected of harboring PCa by distinguishing clinically significant tumors from indolent disease, thereby refining patient selection for biopsy [5]. In this context, considerable efforts have been directed toward optimizing both the pre-biopsy pathway and biopsy techniques without compromising the detection of clinically significant PCa, through the integration of MRI for lesion targeting, the refinement of sampling strategies, and the adoption of safer access routes [6,7,8]. Despite the undeniable contribution of MRI to the contemporary PCa diagnostic pathway, its widespread implementation remains constrained by issues of cost, availability, and radiological expertise, particularly in resource-limited settings [9]. Against this backdrop, novel biomarkers have been introduced into the pre-biopsy setting to improve risk stratification beyond PSA alone, aiming to better identify clinically significant disease and reduce the overdiagnosis of indolent PCa [10]. Within this evolving framework, AS has emerged as the preferred management strategy for patients with low-risk PCa, with the goal of balancing the risk of overtreatment by deferring radical treatment while preserving the opportunity for timely curative intervention in the event of disease progression [11,12]. The following sections review the current evidence underpinning each of these domains, with an emphasis on their integration within a contemporary PCa screening pathway that aims to identify clinically significant PCa and to reduce unnecessary biopsies and overdiagnosis through precise, risk-adapted strategies and by managing through AS those low-risk tumors that are nonetheless detected.

## 2. Methods

We performed a narrative literature review search of PubMed/MEDLINE for original articles using Medical Subject Headings (MeSH) indexes, keyword searches, and publication types from inception until February 2026. The search was limited to articles in English. The search strategy combined MeSH and free-text terms related to prostate cancer screening, including (“prostate cancer” OR “PCa”) AND (“screening” OR “early detection”) AND (“prostate-specific antigen” OR “PSA”) AND (“magnetic resonance imaging” OR “MRI”) AND (“biomarkers” OR “polygenic risk score” OR “risk-adapted screening”). Relevant articles identified in the reference lists of the selected manuscripts were also included. Results for prostate cancer screening strategies from prospective and retrospective studies and randomized controlled trials were reported. The population, intervention, comparison, outcomes, and study design (PICOS) framework was used to define the inclusion criteria [13] (Table 1). Study selection was performed independently by two authors, and discrepancies were resolved through discussion with a third author to minimize selection bias. The following exclusion criteria were applied: abstracts and non-English articles, review articles, meta-analyses, guidelines, case reports, case series, editorials, and book chapters. Given the narrative nature of this review, the aim was not to provide an exhaustive systematic synthesis but rather a clinically oriented and structured overview of the most relevant and contemporary evidence.

## 3. Prostate Cancer Screening Strategies

### 3.1. PSA-Based Screening Approaches

PCa screening has historically relied on serum PSA testing, with the first evidence supporting its clinical utility emerging in the late 1990s [14,15]. The clinical impact of PSA-based screening has been further evaluated in three landmark randomized trials: the European Randomized Study of Screening for Prostate Cancer (ERSPC); the Prostate, Lung, Colorectal, and Ovarian Cancer Screening Trial (PLCO); and the Cluster Randomized Trial of PSA Testing for Prostate Cancer (CAP) [16] (Table 2).

The ERSPC was a multicenter randomized trial conducted across eight European centers to evaluate the impact of PSA-based screening on PCa mortality. The study enrolled approximately 162,000 men aged 55–69 years, who were randomly assigned either to a screening group undergoing PSA testing once every 4 years (every 2 years in Sweden) or to a control group not invited to screening. Prostate biopsy was performed in cases of PSA > 3 ng/mL. The first report, published in 2009, demonstrated a 20% relative reduction in PCa mortality among men invited to screening after a median follow-up of nine years (RR 0.81; 95% CI 0.65–0.98), at the cost of the substantial overdiagnosis of low-risk disease compared with controls (PCa incidence rates of 8.2% vs. 4.8%, respectively, in the screening vs. control arms) [17]. In the contemporary era, where many low-risk screening-detected tumors are managed with AS rather than immediate radical treatment, the trade-offs of screening may be more appropriately interpreted not only in terms of the number needed to treat (NNT) but also in terms of the number needed to diagnose (NND). This distinction is particularly relevant when interpreting historical screening trials, in which treatment was more frequently delivered irrespective of the disease grade, whereas, in current practice, AS has reduced the therapeutic burden associated with the overdiagnosis of indolent PCa. Subsequent analyses with extended follow-up at 11, 13, and 16 years reported an unchanged relative reduction in PCa mortality of 21%, alongside a progressive decrease in the number needed to invite (NNI) (1055 vs. 781 vs. 570, respectively) and the NND (37 vs. 27 vs. 18, respectively) to avert one PCa death [18,19,20]. The recently published 23-year follow-up confirmed a substantial and clinically meaningful 13% relative reduction in PCa mortality in the screening population (RR 0.87; 95% CI 0.80–0.95). Moreover, the NNI and NND were further improved to 456 and to 12, respectively. However, the final analyses also highlighted a higher cumulative incidence of PCa in the screening group compared with controls (14% vs. 12%). When stratified by risk category, most detected cases were classified as low-risk disease, emphasizing the issue of overdiagnosis inherent to PSA-only screening [21]. Overall, the ERSPC trial provided evidence that PSA-based screening reduces PCa-specific mortality over long-term follow-up, although this benefit must be carefully balanced against the risk of overdiagnosis.

The Göteborg-1 randomized population-based screening trial represented the Swedish component of the ERSPC protocol and provided important evidence supporting PSA-based screening. The trial enrolled 20,000 men aged 50–64 years, who were randomized to receive biennial PSA screening or to usual care. A PSA cut-off of between 2.5 and 3 ng/mL was used to select candidates for prostate biopsy. The initial report, with 14 years of follow-up, demonstrated an absolute reduction in PCa mortality of 0.40% (95% CI 0.17–0.64) in the screening group, corresponding to an NNI of 293 and an NND of 12 to avert one death from PCa [22]. After 22 years, the Göteborg-1 trial confirmed a durable reduction in PCa mortality between the screening and control groups (absolute reduction, 0.59%; 95% CI 0-43-0.80), with further improvement in screening efficiency (NNI = 221; NND = 9). The screening benefit was more pronounced in men who started PSA testing before age 60. Despite this, the cumulative incidence of PCa remained higher in the intervention arm compared with controls (18.6% vs. 14.3%, respectively), underscoring the persistent issue of overdiagnosis [23].

The PLCO trial, conducted in the United States, randomized more than 76,000 men aged 55–74 to receive annual screening with PSA for six years combined with DRE for four years or to usual care. A PSA threshold of >4 ng/mL was used to refer participants for prostate biopsy. Extended follow-up analyses at 7, 13, 15, and 17 years consistently demonstrated a higher incidence of PCa in the screening arm, with no statistically significant difference in prostate cancer-specific mortality between the two groups. Specifically, the last report, published in 2019, showed PCa incidence rates of 106 and 101 per 10,000 person-years (RR 1.05; 95% CI 1.01–1.09) and PCa mortality rates of 5.5 and 5.9 per 10,000 person-years (RR 0.93; 95% CI 0.81–1.08) in the screening vs. control groups, respectively. These results have been largely attributed to considerable contamination in the control arm, as more than 80% of men underwent opportunistic PSA testing outside the trial protocol, thus markedly reducing the contrast between the two groups. However, subsequent modeling analyses indicated that, under lower levels of contamination, the PLCO trial would have been consistent, with a 27% to 32% reduction in PCa mortality in favor of screening [24]. Another key finding from PLCO was that screening led to the increased detection of low-grade cancers (defined as Gleason score 2–6; RR 1.17; 95% CI 1.11–1.23) with a concomitant reduction in high-grade cancers (Gleason score 8–10; RR 0.89; 95% CI 0.80–0.99), without a corresponding survival benefit. Finally, secondary analyses conducted within a PLCO subcohort showed that the baseline PSA level strongly predicted the long-term risk for clinically significant PCa (csPCa), supporting the notion that screening can be less frequent or discontinued when PSA levels are <1–2 ng/mL [25].

The CAP trial was the largest randomized study evaluating PSA-based screening to date. Conducted in the United Kingdom between 2001 and 2009, it enrolled over 415,000 men aged 50–69 years. Unlike ERSPC and PLCO, the CAP trial assessed the effect of a single invitation to PSA testing compared with a no-screening approach. Patients with PSA > 3 ng/mL were referred for prostate biopsy. The primary analysis, published in 2018, showed that, after a median follow-up time of 10 years, a single PSA screening invitation did not result in a significant reduction in PCa mortality (RR 0.96; 95% CI 0.85–1.08). This was accompanied by a higher cumulative incidence of PCa in the intervention arm compared to controls (4.3% vs. 3.6%, respectively), as well as the increased detection of low-risk PCa (Gleason score 2–6: 1.7% vs. 1.1%, respectively; difference per 1000 men, 6.11; 95% CI 5.38–6.84) [26]. These findings were mostly confirmed at 15 years of follow-up, suggesting that low-intensity, one-time PSA screening is insufficient to achieve a meaningful reduction in PCa mortality at the population level [27]. Notably, adherence to screening in the intervention was relatively low, with only 36% of invited men undergoing PSA testing. Moreover, patients underwent opportunistic PSA testing outside the study protocol, further reducing the contrast between the two groups [28].

Taken together, the available evidence suggests that PSA-based screening can reduce PCa-specific mortality when applied repeatedly and over long-term follow-up. However, these benefits come at the cost of substantial overdiagnosis and the downstream risk of overtreatment. These limitations highlight the need for more refined, risk-adapted early detection strategies capable of maintaining the mortality benefit of screening while reducing unnecessary diagnoses and interventions.

### 3.2. Digital Rectal Examination: Current Role and Limitations

DRE, in combination with PSA testing, has historically been part of the PCa screening protocol, based on its ability to detect palpable abnormalities suggestive of clinically significant disease. However, the contribution of DRE to the early detection of PCa remains controversial, with conflicting evidence emerging from different randomized trials.

Data from the screening arm of the PLCO trial demonstrated that suspicious DRE, together with elevated PSA levels, was independently associated with an increased risk of csPCa (HR 2.21, 95% CI 1.99–2.44, *p* < 0.001) and PCa-specific mortality (HR 2.54, 95% CI 1.41–4.58, *p* = 0.002) [29]. More recently, a secondary analysis of the PLCO trial showed that abnormal DRE was associated with a 23% higher annual risk of PCa as diagnosis approached (OR 1.230, 95% CI 1.193–1.268), supporting the importance of long-term follow-up with DRE in PCa detection [30].

In contrast, the PROBASE study, a randomized clinical trial conducted in Germany, demonstrated that DRE did not contribute significantly to early PCa detection, with a positive predictive value (PPV) of only 0.87% and a detection rate of 0.03% among 45-year-old participants [31,32]. These results were reinforced by further analyses demonstrating that standalone DRE has poor diagnostic performance and does not improve cancer detection in young men, even in the presence of elevated PSA levels [32]. Similarly, long-term data from a Scandinavian trial evaluating men with PSA levels between 3.0 and 3.9 ng/mL did not support the role of DRE as a screening test [33].

### 3.3. Magnetic Resonance Imaging (MRI) in PCa Screening

In recent years, MRI has been integrated into PCa screening strategies, with the aim of improving the detection of clinically significant disease while reducing overdiagnosis and unnecessary biopsies. MRI can be used either as a first-line test, offered to every participant in the screening program, or as a second-line test, performed only in men with an elevated pre-test risk based on PSA or clinical parameters. This distinction is crucial, as first-line and second-line strategies differ substantially in terms of cost-effectiveness, overdiagnosis rates, and the detection of csPCa. Several trials have investigated the accuracy and feasibility of MRI as part of early detection pathways, highlighting its potential to overcome the limitations of PSA-based approaches (Table 3).

The IP1-PROSTAGRAM study was among the first population-based trials to evaluate the use of MRI as a first-line screening tool for PCa. The analyses showed that non-contrast MRI protocols can detect csPCa more frequently than PSA alone, without increasing the rate of unnecessary biopsies or the overdiagnosis of indolent disease [34]. Similar findings emerged from the Canadian MVP randomized trial, further supporting the role of MRI as a screening strategy. In this study, patients who underwent MRI screening had higher detection rates of PCa (RR 1.89; 95% CI 0.82–4.38, *p* = 0.14) and clinically significant disease (RR 2.77; 95% CI 0.89–8.59, *p* = 0.07) compared with those screened with PSA alone [35]. The VISIONING trial assessed the use of bpMRI as a standalone screening tool, demonstrating a superior ability to detect csPCa. Notably, the use of a PSA threshold of 3 ng/mL would have resulted in missing approximately 40% of patients with ISUP Grade Group (GG) ≥ 2 PCa [36]. Complementing these results, data from the PROSA trial showed a statistically significant increase in the detection of csPCa when bpMRI was used as a first-step screening approach, irrespective of PSA values, compared with the conventional pathway of PSA testing followed by MRI (4.6% vs. 1.8%, respectively; RR 2.6, 95% CI 1.1–6.1, *p* = 0.05). However, the higher number of cancer diagnoses was associated with an estimated additional cost of EUR 2000–3000 per case of csPCa detected [37].

Further refinement of MRI-based screening strategies has been explored in trials combining PSA and MRI in a complementary or sequential manner. Eklund et al. conducted a population-based noninferiority trial involving approximately 50,000 men aged 50 to 74 with PSA level ≥ 3 ng/mL. Among these, 1532 participants met the inclusion criteria and were randomized to either standard biopsy or MRI followed by standard plus targeted biopsy in the case of positive MRI. The proportion of indolent tumors diagnosed was significantly lower in the experimental group compared to the standard biopsy group (4% vs. 12%, difference of −8 percentage points; 95% CI −11 to −5), highlighting the key role of MRI in improving the PCa screening process [38]. The Göteborg-2 trial focused on men with PSA levels between 1.8 and 3 ng/mL, a range traditionally associated with diagnostic uncertainty. The results demonstrated that MRI could identify a non-negligible burden of csPCa within this PSA interval [39]. Two subsequent analyses of the Göteborg-2 cohort by Hugosson et al. evaluated screening strategies including PSA testing followed by MRI, with targeted biopsy only in MRI-positive cases. Both studies highlighted the key role of MRI in accurately identifying men at increased risk of clinically significant cancer, avoiding biopsies in the case of negative MRI and reducing overdiagnosis in men with low-risk disease [40,41]. Additionally, the role of screening with PSA and/or MRI has also been investigated in male carriers of BRCA1/2 gene mutations, given the higher incidence of PCa in this population. The first round of a screening protocol conducted by Segal et al. demonstrated that, in BRCA carriers younger than 55 years, MRI alone without PSA testing provided the highest net benefit for the diagnosis of clinically significant disease (94% of cancer cases detected) [42].

Taken together, these findings suggest that integrating MRI into screening strategies could help to refine early diagnosis and risk stratification in men undergoing screening for PCa. In particular, second-stage MRI-based screening yields higher detection rates of csPCa, likely reflecting the higher pre-test probability of disease among PSA-selected men, while simultaneously mitigating the overdiagnosis of low-risk cancers. Despite heterogeneity in study design, MRI protocols (biparametric versus multiparametric), and target populations, the available evidence suggests that the incorporation of MRI into screening pathways may reduce unnecessary biopsies while improving the overall benefit-to-harm profile of PCa screening.

### 3.4. Emerging Serum and Urinary Biomarkers

Novel biomarkers combining clinical and genetic information may help to refine PCa screening eligibility by improving individual risk estimation for clinically significant disease when incorporated into screening strategies. Among the validated tools currently available are Stockholm3 (STHLM3), the 4Kscore, the Prostate Health Index (PHI), selected urinary biomarkers, and emerging polygenic risk score (PRS)-based approaches. Table 4 provides a structured overview of these biomarkers.

The STHLM3 test integrates six plasma proteins (PSA, free PSA, intact PSA, hK2, MSMB, and MIC1), 232 genetic variants, and clinical variables for PCa risk stratification. Results from the landmark study comparing STHLM3 at a threshold of ≥0.11 combined with MRI and target biopsies versus PSA-based screening at ≥3 ng/mL with systematic biopsies demonstrated a significantly higher AUC for csPCa detection (0.76 [95% CI 0.72–0.80] vs. 0.60 [95% CI 0.54–0.65]). At a threshold of ≥0.15, Stockholm3 maintained equivalent sensitivity while reducing biopsy referrals by 32–55% [43]. External validation in the prospective SEPTA trial, which included racially and ethnically diverse participants, confirmed noninferior sensitivity versus PSA ≥ 4 ng/mL (relative sensitivity 0.95 [95% CI 0.92–0.99]) and nearly three-fold higher specificity (relative specificity 2.91 [95% CI 2.63–3.22]), consistent across all racial and ethnic subgroups [44]. Nine-year follow-up from the STHLM3 cohort further demonstrated that STHLM3 identifies aggressive cancer in men with PSA <3 ng/mL, showing an HR of 1.8 (0.8–3.9; *p* = 0.2) for any BCR and an HR of 8.8 (1.06–72; *p* = 0.044) for high-risk BCR according to the EAU classification [45], allowing for safe biopsy deferral in individuals with low scores (5-year BCR risk 1.5% [0.3–4.8%] [46]. STHLM3 is clinically validated and CE-IVD-certified, with a lifetime microsimulation demonstrating an ICER of EUR 5663/QALY and a 97% probability of cost-effectiveness at a willingness-to-pay value of EUR50,000/QALY versus PSA-alone screening [47].

The 4Kscore integrates four kallikrein markers (total PSA, free PSA, intact PSA, and hK2) with clinical variables to estimate the individual risk of csPCa. Results from a prospective multicenter study demonstrated an AUC 0.82 for csPCa, with the potential to avoid 30–58% of biopsies depending on the risk threshold applied [48]. Evaluated as a reflex test in the Göteborg-2 Biomarker study in 571 men with PSA ≥ 3.0 ng/mL, the 4Kscore demonstrated an AUC of 0.84 (95% CI 0.79–0.89) for intermediate- to high-grade PCa, with an NPV of 99% (95% CI 97–100%) [49]. The ProScreen randomized trial validated a sequential PSA, 4Kscore, and MRI-directed biopsy strategy, with only 2.7% of screened participants requiring biopsy [50]. However, long-term data showed that the 4Kscore did not significantly outperform total PSA alone for high-grade cancer prediction, with the benefit restricted to men under 60 with PSA ≥ 2 ng/mL [51]. The 4Kscore is clinically validated and FDA-approved but not CE-IVD-certified for the European market. A budget impact model demonstrated net savings of USD 169 million (15.6% of total costs) and USD 1694 per patient versus a biopsy-for-all strategy in men with elevated PSA [52].

The PHI combines total PSA, free PSA, and [-2]proPSA into a single validated score for men with PSA 4–10 ng/mL and non-suspicious DRE. The PHI output is stratified into three clinically actionable zones: scores below 27 are associated with a low probability of csPCa (~91% of biopsied men) and scores above 55 indicate a 76% likelihood of clinically significant disease, while the intermediate range (27–55) defines a gray zone where additional risk stratification is most relevant [53]. The multicenter validation by Catalona et al. demonstrated PHI specificity of 16.0% versus 8.4% for %fPSA at 95% sensitivity (*p* = 0.015) and a superior AUC for overall cancer detection (0.703 vs. 0.648; *p* = 0.004), with PHI ≥ 55 associated with a 1.61-fold increased risk of ISUP GG ≥ 2 [54]. Guazzoni et al. confirmed the PHI as the most accurate individual predictor at biopsy (AUC 0.756 vs. 0.53 for tPSA; *p* < 0.001) in 268 men referred for initial extended biopsy, improving the multivariate accuracy from 72% to 83% in the PSA 4–10 ng/mL subgroup [55]. Similarly, White et al. demonstrated that the use of PHI testing in clinical practice led to a 24% absolute reduction in prostate biopsies performed and influenced physician management in 73% of cases [56]. The PHI is clinically validated, FDA-approved, and CE-IVD-certified, with microsimulation modeling demonstrating an 11% improvement in cost-effectiveness versus PSA-alone screening [57].

PRS-based strategies represent a promising approach for risk-adapted PCa screening. BARCODE1 evaluated a 130-SNP PRS in 6393 men aged 55–69, referring men at the ≥90th centile (11.7% of genotyped participants) for mpMRI and transperineal biopsy irrespective of the PSA level. Among 468 biopsies performed, 40% were PCa-positive, 55.1% indicated csPCa, and 63.1% of cancer cases were detected in men with PSA ≤ 3.0 μg/L, with an estimated overdiagnosis rate of 20.8% [58]. Rare pathogenic germline variants (PGVs) in high-penetrance genes represent a complementary dimension of inherited risk. The IMPACT study reported a significantly higher clinically significant cancer incidence in BRCA2 carriers versus non-carriers (3.1% vs. 1.3%; *p* = 0.039), with diagnosis at a younger age (61 vs. 64 years; *p* = 0.026) and higher proportions of NCCN intermediate–unfavorable/high-risk tumors in both BRCA2 (65% vs. 32%; *p* = 0.029) and BRCA1 carriers (56% vs. 18%; *p* = 0.0017), and with pathological upgrading at radical prostatectomy occurring exclusively among PGV carriers [59]. These findings support EAU guideline recommendations for annual PSA screening in BRCA2 carriers from age 40, with consideration in BRCA1 carriers.

Several urinary and serum-based molecular assays have been developed to refine biopsy decision-making in men with elevated PSA. SelectMDx is a urinary mRNA-based assay that quantifies the post-DRE expression of HOXC6 and DLX1 combined with clinical variables to generate a risk score for csPCa. In a prospective multicenter study of 163 biopsy-naïve patients with PSA 3–10 ng/mL, SelectMDx did not significantly outperform mpMRI alone in the detection of csPCa, with an AUC of 0.63 (95% CI 0.56–0.71) versus 0.63 for mpMRI alone (95% CI 0.57–0.70). SelectMDx showed sensitivity of 76.9% (95% CI 63.2–87.5%) and an NPV of 82.1% (95% CI 70.8–90.4%). Combination with mpMRI improved the NPV to 92.6% (95% CI 75.7–99.1%) and the AUC to 0.75 (95% CI 0.66–0.83) [60], although its incremental value within an MRI-first diagnostic pathway remains uncertain [61]. SelectMDx is clinically validated and CE-IVD-certified. A decision-analytical model demonstrated savings of EUR 128/patient and +0.025 QALYs versus PSA-alone standard of care [62]. Prostate Cancer Antigen 3 (PCA3) is a prostate-specific long non-coding RNA that is measurable in post-DRE urine and was among the first molecular assays approved to aid repeat biopsy decisions in men with elevated PSA and a prior negative biopsy [63]; however, as a standalone test, its clinical utility remains uncertain [64]. Today, PCA3 is mainly incorporated within multiplex urinary assays. MyProstateScore (MPS) combines PCA3 and TMPRSS2:ERG with serum PSA into a single risk score for pre-biopsy triage [65]. Its 18-gene iteration (MPS2) adds additional high-grade-associated transcripts to enhance discrimination for clinically significant disease. External validation by Tosoian et al. [66] demonstrated an AUC of 0.81 (95% CI 76.9–84.6) for GG ≥ 2 cancer, outperforming PSA alone (AUC 0.60; 95% CI 54.7–64.6), the PHI (AUC 0.77; 95% CI 73.0–81.3), and the original MPS (AUC 0.74; 95% CI 69.4–78.0). At 95% sensitivity for GG ≥ 2 disease, MPS2 reduced unnecessary biopsies by 35% in the initial biopsy population and 46% in the repeat biopsy setting, with NPVs of 95–99% for GG ≥ 2. MPS2 is clinically validated and available in the US as a CLIA-certified laboratory-developed test. The ExoDx Prostate IntelliScore (EPI), a non-DRE voided urine exosome assay measuring PCA3, ERG, and SPDEF, achieved an AUC of 0.74 in a prospective cohort of 519 men with PSA 2–10 ng/mL, with 92% sensitivity and a 91% NPV at the validated cut-off, allowing 27% of biopsies to be avoided; its incremental value over the standard of care and its role within an MRI-first pathway remain to be prospectively defined [67]. The EPI is clinically validated. It is currently CE-IVD-certified in Europe and has been granted FDA Breakthrough Device Designation, although formal FDA approval remains pending.

Overall, biomarker-based risk-adapted models may offer a more tailored approach than PSA-only strategies. A central clinical issue remains how to combine these tools to improve the detection of clinically significant disease while minimizing overdiagnosis, the procedural burden, and healthcare costs across different settings.

### 3.5. Biopsy Strategies Following Positive Screening Tests

A positive screening test does not establish a diagnosis; thus, histological verification through prostate biopsy remains the diagnostic gold standard. Prostate biopsies are generally well tolerated and associated with a low overall complication rate. Potential adverse events include hematuria, hematospermia, urinary retention, rectal bleeding, urinary tract infections, and, less commonly, sepsis. Considerable efforts have been directed toward optimizing the diagnostic pathway prior to biopsy to reduce unnecessary invasive procedures and their associated side effects, particularly infectious complications, without compromising cancer detection.

The PROMIS trial, a prospective multicenter paired-cohort diagnostic accuracy study enrolling 740 biopsy-naïve men, assessed the diagnostic accuracy of mpMRI and TRUS-guided biopsy against transperineal template mapping biopsy as the reference standard, with all men undergoing all three procedures in a blinded sequential design. For csPCa, mpMRI was significantly more sensitive than TRUS biopsy (93% [95% CI 88–96%] vs. 48% [95% CI 42–55%]; *p* < 0.0001), with a higher NPV (89% [95% CI 83–94%] vs. 74%; *p* < 0.0001). Negative mpMRI would have allowed 27% of patients to safely avoid biopsy, with an estimated 5% reduction in overdiagnosis [5]. The PRECISION trial (n = 500; 25 centers, 11 countries) confirmed that mpMRI-targeted biopsy detected more clinically significant cancer than standard 12-core TRUS biopsy (38% vs. 26%; adjusted difference 12%; *p* = 0.005), with less indolent disease (9% vs. 22%; *p* < 0.001) and fewer cores (median 4 vs. 12) [6], establishing the foundation for incorporating pre-biopsy mpMRI into international guidelines.

Some trials have investigated the debated choice between transrectal and transperineal prostate biopsy approaches. The TRANSLATE trial (n = 1126 biopsy-naïve patients) demonstrated superior GG ≥ 2 detection with transperineal versus transrectal biopsy (60.1% vs. 54.4%; OR 1.32, 95% CI 1.03–1.70; *p* = 0.031), with fewer infectious complications requiring hospitalization at 35 days (2 [<1%] vs. 9 [2%]) [8]. The PREVENT trial (n = 658) confirmed no infections in the transperineal arm versus 1.4% in the transrectal arm (difference −1.4%; *p* = 0.059), with comparable csPCa detection [68]. In contrast, the PERFECT trial (n = 270) did not demonstrate equivalence between routes overall (47.2% vs. 54.2%; *p* = 0.623), with a route-by-location interaction favoring the transrectal approach for posterior lesions [69].

Perilesional biopsy is emerging as a novel prostate biopsy technique aimed at improving the detection of clinically significant disease while potentially reducing the number of biopsy cores required. This approach concentrates sampling within a circumferential margin surrounding the MRI-defined region of interest (ROI). Brisbane et al. demonstrated that 90% (95% CI 89–91%) of csPCa cores were located within a 10 mm radius of the lesion, and 18% of patients had csPCa diagnosed exclusively outside the ROI; the penumbra width capturing 90% of csPCa was inversely related to the MRI grade (5 mm for PI-RADS 5, up to 16 mm for PI-RADS 3) [70]. The MIRAGE study prospectively confirmed that perilesional sampling added +3.6% GG ≥ 2 yield (*p* < 0.001) at the cost of only +1% GG1, while distant systematic cores provided a minimal incremental yield, supporting the omission of distant sampling when a targeted-plus-perilesional strategy is applied [71].

Collectively, these data highlight the progressive refinement of prostate biopsy strategies aimed at maximizing the detection of csPCa while minimizing unnecessary procedures and associated morbidity.

### 3.6. The Role of Active Surveillance (AS)

A substantial proportion of cancer cases detected through population-based screening are classified as low-risk disease. Overtreatment may expose men to the morbidities of radical prostatectomy or radiotherapy, including urinary incontinence, erectile dysfunction, and radiation-induced sequelae.

AS is a structured monitoring strategy designed to defer radical treatment in men with low-risk PCa while preserving the option for curative intervention if disease progression is demonstrated. Eligibility has historically been restricted to men with GG1 disease (PSA < 10 ng/mL, GG1 on systematic biopsy, cT ≤ 2a, PSA-D < 0.15 ng/mL/cc), with contemporary protocols increasingly incorporating selected GG2 patients with limited MRI-visible disease and a low tumor burden at biopsy. Other histological features that preclude AS include a cribriform pattern or intraductal carcinoma. Contemporary AS protocols include PSA measurement every 3–6 months, annual clinical assessment with DRE, and serial mpMRI assessed using the Prostate Cancer Radiological Estimation of Change in Sequential Evaluation (PRECISE) scoring system [72]. Confirmatory biopsy is recommended within 12–18 months after diagnosis, whereas the timing and frequency of subsequent surveillance biopsies remain controversial. Triggers for radical treatment include pathological upgrading to GG ≥ 2 (or GG ≥ 3 in selected GG2 patients), significant radiological progression on serial mpMRI, or patient preference [45]. Foundational evidence for AS is derived from historical cohorts in which the 10- and 15-year cause-specific survival were 98.1% and 94.3%, respectively, with only 15 prostate cancer deaths (1.5%) [11,12]. PRIAS demonstrated 77.3% treatment-free survival at 2 years, with the PSA density and number of positive cores as the strongest independent predictors of reclassification [73]. The ASIST randomized controlled trial demonstrated that baseline MRI reduced AS failures by approximately half at 2 years (19% vs. 35%; *p* = 0.017) and significantly decreased grade progression (9.9% vs. 23%; *p* = 0.048) [74,75], establishing that MRI improves the quality of patient selection and reduces undetected occult aggressive disease over time.

The feasibility of AS for GG2 PCa remains a debated frontier: the Memorial Sloan Kettering series reported treatment-free survival of 61% (95% CI 52–70%) at 5 years and 49% (95% CI 37–60%) at 10 years, with no distant metastases or prostate cancer deaths at a median of 3.1-year follow-up; diagnostic PSA (HR 1.08; *p* = 0.003) and total millimeters of cancer at diagnosis (HR 1.07; *p* = 0.021) were independently associated with treatment discontinuation [76,77]. The Toronto/Sunnybrook experience showed that intermediate-risk disease carried a significantly higher risk of metastasis versus low-risk disease (HR 3.14, 95% CI 1.51–6.53), with 15-year metastasis-free survival of 82% for GG2 versus 95% for GG1 [78]. Extended follow-up from the Göteborg-1 trial, conducted in the pre-MRI era using sextant biopsies and without a predefined AS protocol, confirmed PC-specific survival of 94% (95% CI 91–98%) at 25 years, with only 14 PC-related deaths. Stratified by risk group, PC-specific survival was 99% for very-low-risk, 92% for low-risk, and 85% for intermediate-risk disease at 24 years. However, the intermediate-risk group accounted for 9 of 14 PCa-related deaths, and GG ≥ 2 was the strongest independent predictor of failure on multivariable analysis (HR 3.12; 95% CI 1.59–6.13; *p* < 0.001), with failure-free survival at 19 years of only 55% versus 85% for very-low-risk disease [79].

These data confirm that deferred treatment is oncologically safe in carefully selected GG1 and GG2 patients while underscoring the fact that intermediate-risk disease warrants more intensive monitoring and that the window for safe AS in this subgroup, while real, is considerably narrower than in GG1 disease.

## 4. Discussion

Prostate cancer screening has undergone substantial evolution over the past three decades. Early screening strategies based primarily on PSA testing demonstrated a measurable reduction in prostate cancer-specific mortality in large randomized trials [17,23]. However, these benefits were accompanied by the substantial overdiagnosis of indolent tumors and the consequent risk of overtreatment [21,23,26,27]. The extent of overdiagnosis was particularly evident in the consistent excess of low-risk disease detected across trials, raising concerns regarding the overall benefit–harm balance of population-based screening [24,25]. In response to these limitations, contemporary screening strategies are increasingly incorporating more individualized and risk-adapted elements, moving beyond a one-size-fits-all approach. The evidence reviewed in this article highlights how several diagnostic innovations have contributed to reshaping the PCa screening landscape.

The integration of MRI into the diagnostic pathway has been associated with the improved detection of clinically significant disease, along with reductions in biopsy rates ranging from approximately 25% to 50%, depending on the study design and screening pathway [34,35,36,37]. Despite these advantages, the diagnostic performance of mpMRI remains highly dependent on radiological expertise and may be affected by inter-reader variability, as well as by false-negative findings, particularly in small-volume tumors that may still harbor clinically relevant disease.

In parallel, the development of novel serum, urinary, and genomic biomarkers has provided additional tools for pre-biopsy risk stratification, allowing clinicians to better estimate the individual probability of csPCa before proceeding with invasive diagnostic procedures. Among these, validated serum-based tools such as Stockholm3, the 4Kscore, and the PHI have demonstrated consistent improvements in specificity over PSA alone, with reported reductions in biopsy rates of between 24% and 58%, depending on the thresholds and populations, without compromising the detection of clinically significant disease [43,56]. Urinary assays further extend this framework by incorporating tumor-specific molecular information that cannot be captured through clinical variables alone [60,67]. Looking ahead, prospective data from BARCODE1 support the potential role of polygenic risk scores in refining individualized screening strategies [58], while the recently launched TRANSFORM platform trial is evaluating PRS-guided MRI screening on a national scale across multiple intervention strategies, with the potential to refine how men are selected for screening in the near future [80]. Although mpMRI is already part of contemporary clinical practice and its clinical and economic value has been demonstrated, the situation is different for biomarkers. Despite their potential to refine risk stratification and reduce unnecessary biopsies, the implementation of serum, urinary, and genomic biomarkers remains limited by costs, reimbursement policies, and variable availability across healthcare systems. Therefore, their broader integration into routine clinical practice remains to be fully established [81,82].

Improvements in biopsy techniques have further optimized the diagnostic pathway. The adoption of MRI-targeted biopsy approaches, together with the increasing use of the transperineal approach, has further improved the diagnostic yield for clinically significant disease while minimizing procedure-related morbidity [6,8,68,69,70,71]. Importantly, the evolution of screening has been paralleled by major advances in disease management. The widespread adoption of active surveillance for low-risk prostate cancer has substantially mitigated the historical problem of overtreatment, allowing many patients to safely defer radical therapy while maintaining excellent oncological outcomes [11,12,73].

This narrative review has inherent limitations that should be acknowledged. The absence of a pre-registered protocol and a standardized selection algorithm may expose the synthesis to selection and publication bias, as studies with positive findings may be overrepresented. The heterogeneity of the included studies in terms of populations, PSA thresholds, and outcome definitions precluded a formal quantitative synthesis. Moreover, despite a search updated to February 2026, the rapidly evolving landscape of PCa early detection means that some recent evidence may not have been captured. Finally, most available evidence comes from high-income countries, and the implementation of MRI- and biomarker-based screening pathways in low-resource settings may be hampered by limited access to MRI scanners, radiological expertise, and advanced biomarkers, as well as by competing healthcare priorities.

## 5. Conclusions

The future of PCa screening lies in a more personalized and harm-aware approach. Beyond PSA-based screening, the integration of MRI and novel biomarkers may improve risk stratification and help to better identify clinically significant disease, although further prospective studies with mature mortality outcome data are needed. At the same time, the widespread adoption of AS has been proven to optimize the risk–benefit balance by reducing the overtreatment of indolent disease.

## Figures and Tables

**Table 1:** Population, intervention, comparison, outcomes, and study design (PICOS) framework and search strategy.

**Clinical Question**	To Summarize the Evidence Regarding Contemporary Strategies for Prostate Cancer Screening and Early Detection
**Population**	Asymptomatic men eligible for PCa screening in the general population or risk-stratified populations
**Intervention**	Screening strategies: PSA testing, MRI-based screening pathways, reflex biomarkers, and polygenic risk-adapted screening approaches
**Comparison**	No screening, opportunistic screening, alternative screening strategies, or different screening intervals/thresholds
**Outcomes**	Overdiagnosis rates, biopsy rates, CSM, OCM, and screening-related harms
**Study design**	Prospective and retrospective studies and randomized controlled trials
**Databases searched**	PubMed
**Search terms used (including MeSH and keyword text)**	(prostate cancer) OR (PCa) AND (screening) OR (early detection) AND (prostate-specific antigen) OR (PSA) AND (magnetic resonance imaging) OR (MRI) AND (risk-adapted screening) OR (polygenic risk score) AND (biomarkers) AND (mortality) OR (cancer-specific mortality)
**Manual search**	Relevant citations in identified articles and additional manual examination of articles published in peer-reviewed journals
**Eligibility criteria**	English full-text articles published up to February 2026, evaluating prostate cancer screening strategies in asymptomatic men, including PSA-based, imaging-based, or risk-adapted screening approaches
**Exclusion criteria**	Review articles, meta-analyses, guidelines, case reports, case series, editorials, and book chapters; preclinical studies not involving humans; studies not reporting screening-related outcomes

**Abbreviations:** Prostate cancer (PCa); prostate-specific antigen (PSA); magnetic resonance imaging (MRI); cancer-specific mortality (CSM); other-cause mortality (OCM).

**Table 2:** PSA-based clinical trials.

Trial	**A 16-Year Follow-Up of the European Randomized Study of Screening for Prostate Cancer** **(ERSPC)**	**European Study of Prostate Cancer Screening—23-Year Follow-Up** **(ERSPC)**	**Mortality Results from the Göteborg Randomized Population-Based Prostate Cancer Screening Trial:** **Göteborg-1**	**Prostate Cancer Screening in the Randomized Prostate, Lung, Colorectal, and Ovarian Cancer Screening Trial: Mortality Results After 13 Years of Follow-Up** **(PLCO)**	**The Cluster Randomized Trial of PSA Testing for Prostate Cancer (CAP)**
Population	European multicenter population-based randomized screening trial, 162,236 men, 55–69 years old	European multicenter population-based randomized screening trial, 162,236 men, 55–69 years old	Sweden, prospective randomized population-based prostate cancer screening trial, 20,000 men aged 50 to 64 years	United States, multicenter, randomized two-arm trial, 76,685 men aged 55–74 years	United Kingdom, multicenter randomized trial, 419,582 men aged 50 to 69 years
Year of publication	2019	2025	2010	2016	2018, with update in 2024
Intervention	Repeated PSA screening	Repeated PSA screening	Biennial PSA screening	Annual PSA + DRE	Single PSA invitation
Comparison	No screening invitation	No screening invitation	No screening invitation	Usual care (sometimes including opportunistic screening)	No invitation
Endpoints	Prostate cancer-specific mortality	Prostate cancer-specific mortality	Primary endpoint was prostate-cancer specific mortality, analyzed according to intention-to-screen principle	Prostate cancer-specific mortality	Prostate cancer-specific mortality
Key results	0.16% absolute risk difference (95% CI 0.07–0.24)	0.22% absolute risk difference (95% CI 0.10–0.34)	PSA-based screening: increased ↑ PCa incidence (12.7% vs. 8.2%; HR 1.64); ↓ PCa mortality (RR 0.56, 95% CI 0.39–0.82); absolute risk reduction 0.40% at 14 yrs; RR 0.44	No mortality benefit	No significant reduction in PCa mortality at 10 years (RR 0.96; 95% CI 0.85–1.08); modest reduction at 15 years (RR 0.92; 95% CI 0.85–0.99)
Follow-up	16 years	23 years	18 years	13 years	15 years

**Table 3:** MRI-based clinical trials.

Trial	**Population-Based Prostate Cancer Screening with Magnetic Resonance Imaging or Ultrasonography:** **The IP1-PROSTAGRAM Study**	**Prostate MRI Versus PSA Screening for Prostate Cancer Detection:** **The MVP Study**	**Primary Noncontrast Magnetic Resonance Imaging for Prostate Cancer Screening: A Randomized Clinical Trial (PROSA)**	**Prostate Cancer Screening with PSA and MRI Followed by Targeted Biopsy Only** **(Göteborg-2)**	**MRI-Targeted or Standard Biopsy in Prostate Cancer Screening (STHLM3-MRI)**
Population	United Kingdom, prospective, population-based, blinded cohort study, 2034 men aged 50 to 69 years	Canada, single-center, phase 3, randomized open-label controlled trial, 525 randomized men aged ≥ 50 years	Italy, single-center, randomized controlled trial, 759 men aged 49–69 years	Sweden, population-based, randomized screening trial, 17,980 men aged 50 to 60 years	Sweden, population-based, screening-by-invitation randomized trial, men aged 50–74 years; among screened men, 1532 men with PSA ≥ 3 ng/mL were randomized: 929 to the experimental arm and 603 to the standard biopsy arm
Year of publication	2021	2022	2025	2022	2021
Intervention	MRI-first screening, ultrasonography, or PSA test	bpMRI	bpMRI regardless of PSA level	PSA → MRI → targeted biopsy	PSA ≥ 3 ng/mL → biparametric MRI → if MRI-positive (PI-RADS 3–5), targeted biopsy plus standard systematic biopsy; in men with negative MRI, biopsy was generally omitted, except for those with very high Stockholm3 risk (≥25%)
Comparison	-	PSA testing	bpMRI only if PSA ≥ 3 ng/mL (or 2.5 ng/mL with a family history)	PSA → systematic biopsy	PSA ≥ 3 ng/mL → standard systematic TRUS-guided biopsy
End points	Proportion of men with positive MRI or ultrasonography or PSA test; key secondary outcomes: number of clinically significant prostate cancer cases	Presence of adenocarcinoma on prostate biopsy	Clinically significant prostate cancer detection; secondary outcomes: overall PCa detection, benefit–harm metrics, and cost-effectiveness from a healthcare payer perspective	Primary outcome: clinically insignificant prostate cancer; secondary outcome: clinically significant prostate cancer; safety was also assessed	Primary endpoint: detection of csPCa (ISUP ≥ 2); secondary endpoints: detection of clinically insignificant PCa (ISUP 1), benign biopsy findings, ISUP 3 cancers, and serious adverse events after biopsy
Key results	MRI-first (PI-RADS ≥ 4): ↑ significant PCa detection vs. PSA with similar biopsy rate; US not superior to PSA	MRI vs. PSA: ↓ biopsy recommendation (RR 0.52, 95% CI 0.33–0.82); cancer detection 63% vs. 29%; significant PCa 73% vs. 50%; results are preliminary due to early study cessation	MRI-first: higher biopsy (10.8% vs. 5.2%) and csPCa detection (4.6% vs. 1.8%; RR 2.6, 95% CI 1.1–6.1); better benefit–harm profile; higher costs	Experimental strategy: ↓ clinically insignificant PCa (0.6% vs. 1.2%; RR 0.46, 95% CI 0.33–0.64); no significant difference in csPCa (RR 0.81, 95% CI 0.60–1.10); missed csPCa cases were low-volume and intermediate-risk and managed with active surveillance; serious AEs < 0.1%	MRI-based screening was noninferior to standard biopsy for detection of csPCa: 21% vs. 18% (difference +3%, 95% CI −1 to 7; *p* < 0.001 for noninferiority); it also reduced detection of insignificant cancer: 4% vs. 12% (difference −8%, 95% CI −11 to −5). Biopsies were performed less often with the MRI strategy: 36% vs. 73%; benign biopsy findings were also lower: 11% vs. 43%.

**Table 4:** Serum and urinary biomarkers.

Test	**STHLM3**	**4Kscore**	**PHI**	**SelectMDx**	**MPS2**	**EPI (ExoDx)**
Type	Serum	Serum	Serum	Urine	Urine	Urine
Status	Validated	Validated	Validated	Validated	Validated	Validated
Europe (CE-IVD)	Approved	Not approved	Approved	Approved	Not approved	Approved
USA (FDA)	Not approved	Approved	Approved	Not approved	CLIA-LDT	CLIA-LDT, BTD 2019
Recommended use	Pre-biopsy triage in men with PSA ≥ 3 ng/mL; detection of aggressive cancer in men with PSA < 3 ng/mL	Reflex test in men with PSA ≥ 3 ng/mL; sequential triage with MRI in screening strategies	Men with PSA 4–10 ng/mL and non-suspicious DRE; risk stratification into low (<27), gray zone (27–55), and high probability (>55) categories	Pre-biopsy triage in biopsy-naïve men with PSA 3–10 ng/mL; incremental value when combined with mpMRI (NPV 92.6%)	Pre-biopsy triage (initial and repeat biopsy); superior to PSA, PHI, and original MPS for GG ≥ 2 detection (AUC 0.81); reduces unnecessary biopsies by 35–46%	Men with PSA 2–10 ng/mL prior to biopsy; non-DRE voided urine sample; AUC 0.74, sensitivity 92%, NPV 91%
Cost- effectiveness	ICER EUR 5663/QALY; 97% probability of cost-effectiveness at WTP EUR 50,000/QALY vs. PSA-alone screening	Net savings of USD 169 million (−15.6% total costs); −USD 1694/patient vs. biopsy-for-all strategy	+11% improvement in cost-effectiveness vs. PSA-alone screening in microsimulation modeling	Savings of EUR 128/patient and +0.025 QALYs vs. PSA-alone standard of care	No pharmacoeconomic analysis available	No pharmacoeconomic analysis available

**Abbreviations:** BTD = FDA Breakthrough Device Designation; formal FDA approval pending. CLIA-LDT = CLIA-certified laboratory-developed test, not FDA-cleared.

## Data Availability

No new data were created or analyzed in this study.
